# Comparing the Efficacy of Multiple Drugs Injection for the Treatment of Hypertrophic Scars and Keloid: A Network Meta-Analysis

**DOI:** 10.1007/s00266-022-03163-4

**Published:** 2022-12-19

**Authors:** Wenhao Wu, Yang Zhao, Yuxuan Chen, Aimei Zhong

**Affiliations:** Department of Plastic Surgery, Union Hospital, Tongji Medical College, Huazhong University of Science and Technology, Wuhan, 430022 PR China

**Keywords:** Pathological scar, Keloid, Hypertrophic scar, Network meta-analysis, Efficacy

## Abstract

**Background:**

There is no consensus regarding the choice of injected drugs for pathological scars. Although the clinical efficacy of different drug treatments was shown in many randomized controlled trials, the efficacies of many drugs are inconsistent. Therefore, this study aimed to determine how different effective drugs are for treating pathological scars. It is anticipated that the study findings may serve as guidelines for plastic surgeons.

**Methods:**

Relevant literature was extracted from the following databases Cochrane Library, Embase, PubMed, Web of Science, CNKI, Weipu, and Wanfang until June 2022, such as randomized clinical trials (RCTs) evaluating different injected drugs for the treatment of pathological scars, including BTA, TAC, 5-Fu, VER, and BLE.

**Results:**

This network meta-analysis of 1539 patients from 23 articles revealed that the most effective treatment for a pathological scar was TAC + BTA. The effective rate of TAC + BTA combination therapy was significantly different from that of the BTA, TAC, 5-Fu, VER, and BLM monotherapies. TAC+5-FU was more effective than TAC, 5-FU, VER, or BLM alone, and BTA was more effective than both TAC and 5-Fu. The effectiveness of VER and BLM was the same, but both were better than TAC and 5-Fu. No big differences were found between any of the other local injection therapies.

**Conclusions:**

According to this network meta-analysis, a combination of keloid and hypertrophic scar injection treatment is recommended, especially BTA+TAC. However, this network meta-analysis has some limitations and must be further verified by larger samples and higher quality RCTs.

**Level of Evidence III:**

This journal requires that authors assign a level of evidence to each article. For a full description of these Evidence-Based Medicine ratings, please refer to the Table of Contents or the online Instructions to Authors www.springer.com/00266

## Introduction

In plastic surgery, pathological scars like keloids and hypertrophic scars are considered illnesses. The pathological scar develops when fibroblasts proliferate uncontrollably within the wound and the deposition of an abundance of collagen fibers. This pathological change occurs after tissue injury healing [1, 2]. The skin surface is thicker than regular skin and is frequently characterized by pain, itching, swelling, and discomfort. Additionally, it frequently causes a significant load on people's mental and pathological health [3]. Though keloids and hypertrophic scars can occur anywhere on the body, there are typically found on the front of the chest, the upper arms, the shoulder, the back, the earlobe, the belly, and the joints [4]. It remains unclear what causes keloids and hypertrophic scars [5]. Thus, there is no consensus on how best to manage this disease.

The primary treatments for pathological scars include surgery, radiotherapy, and drug injections [6, 7]. The local drug injections for pathological scar treatment typically include botulinum toxin A (BTA), triamcinolone acetonide (TAC), 5-fluorouracil (5-Fu), verapamil (VER), and bleomycin (BLM) [7], but there are disputes about the choice and efficacy of these drugs. Therefore, how to effectively treat keloids is still a key clinical difficulty [8]. Our findings may help guide physicians toward more effective scar treatments.

## Methods

### Search Strategy

The Cochrane Library, Embase, PubMed, Web of Science, CNKI, Weipu, and Wanfang databases were searched for randomized controlled trials (RCTs) on the therapeutic efficacy of pathological scar up until June 2022. The terms "keloid" or "hypertrophic scar" were used in association with "Triamcinolone Acetonide" or "Botulinum Toxin Type A" or "5-fluorouracil" or "Verapamil" or "Bleomycin" to retrieve relevant articles. Moreover, to prevent the loss of relevant data, the reference list of the identified RCTs was manually examined for potentially relevant papers.

### Inclusion Criteria

These studies were RCTs using only topical drug injections for hypertrophic scars or keloids with BTA, TAC, 5-FU, VER, and BLM; the subject was any patient with hypertrophic scars or keloids, and study outcomes included the treatment efficacy rate (effective patient number divided by the total patient number).

### Exclusion Criteria

Repeated literature; non-randomized controlled studies; other methods or drugs to treat hypertrophic scar or keloid; inability to extract outcome data; animals or cells experiments; evaluation indicators in clinical studies do not include effective rate.

### Quality Assessment

The data were extracted in a standardized format by two reviewers working independently using the data extraction form. First, the duplicates were eliminated, then a preliminary screening was performed by reading the article titles and abstracts. Finally, the full text of the included studies was downloaded. When there was disagreement, the matter was taken to a third person for analysis and resolution. The extracted data comprised the author's name, the region the study was conducted in, the year it was published, the number of males to females in the sample, the average age of those who took part in the study, the therapeutic approach, follow-up, and outcome assessment.

### Statistical Analysis

The Stata16.0 software was used to analyze the data using the risk ratio (RR) and 95% CI. SUCRA were plotted, with the area under the SUCRA proportional to the therapeutic effect of the medication tested, and the efficacy of various treatment measures may be predicted based on the area under the curve. In addition, the funnel plot was used to determine the extent of publication bias in the selected papers.

## Results

### Literature Selection

**Fig 1.**
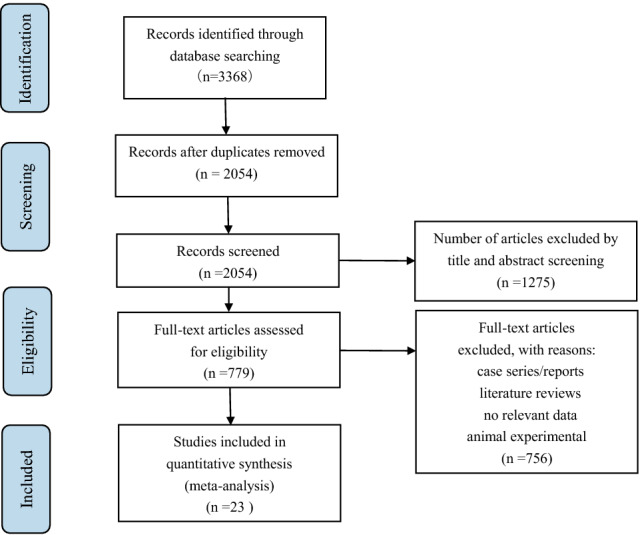
PRISMA flow diagram. PRISMA, Preferred Reporting Items for Systematic Reviews and Meta-Analyses

The relevant articles were selected as shown in Fig. 1. In total, 3368 papers were screened, and 23 were chosen for further analysis [9–31]. There were 2054 studies after removing duplicates, 779 articles were screened by reading titles and abstracts, of which 65 articles were not RCT, 584 articles were for other drugs or treatment modalities, 35 articles were for animal or cell experiments, and 72 articles had no relevant data. In the end, 23 trials with 1539 participants were chosen for this analysis.

The fundamental characteristics that were considered for the meta-analysis are outlined in Table 1. The earliest included study was conducted in 2006, while the most recent was in 2022. The clinical follow-up period lasted from three to twenty-two months. Two articles conducted a direct comparison of the efficacy of BTA and TAC; one article conducted a direct comparison of the efficacy of BTA and 5-Fu; three articles directly compared the efficacy of TAC and 5-Fu; four articles compared the effectiveness of TAC and VER; two articles compared the effectiveness of TAC and BLM; one article compared the efficacy of BTA+TAC and TAC alone; five articles compared the effectiveness of TAC+5-Fu and TAC alone; one article compared the efficacy of TAC+5-Fu and BLM alone; one article compared the efficacy of TAC, 5-Fu and VER; three articles compared the efficacy of BTA alone, TAC alone and BTA+TAC. In terms of the distribution of the population, six studies were conducted in India, five in China, four in Egypt, three in Iran, three in Pakistan, one in Thailand, and one in Finland. The Patient and Observer Scar Assessment Scale (POSAS), the Vancouver Scar Score (VSS), and the Visual Analog Scale (VAS) were used to evaluate the drugs’ effectiveness.

**Table 1:** Characteristics of the 23 included studies

No.	Author	Region	Year	Sex (male/female)	Mean age (range)	Therapeutic method	Follow-up	Outcomes measurement
1	Asilian A [9]	Iran	2006	15/25	23.4 25.3	TAC vs. TAC+5-Fu	12w	POSAS
2	Darougheh A [10]	Iran	2009	40	23.4 25.2	TAC vs. TAC+5-Fu	12w	POSAS
3	Saha AK [11]	India	2012	44	32.96 34.7	TAC vs. 5-Fu	12m	POSAS
4	Khan MA [12]	Pakistan	2014	65/85	29.93 28.96	TAC vs. TAC+5-Fu	12w	POSAS
5	Payapvipapong K [13]	Thailand	2014	10/16	29.8 38.4	TAC vs. Bleomycin	12w	POSAS
6	Hietanen KE [14]	Finland	2019	43	41 46.96	TAC vs. 5-Fu	6m	POSAS
7	Khalid FA [15]	Pakistan	2019	108	31.22 27.67	TAC vs. TAC+5-Fu	22m	POSAS
8	Khan HA [16]	Pakistan	2019	64/100	33 32	TAC vs. Bleomycin	24w	POSAS
9	Ismail SA [17]	Egypt	2020	50	31.20 30.24	BTA vs. 5-Fu	3m	POSAS
10	Sadeghinia A [18]	Iran	2012	40	45 43.36	TAC vs. 5-Fu	44w	POSAS
11	Aggarwal A [19]	India	2018	31	18-50	TAC vs. Verapamil	21w	VSS, VAS
12	Albalat W [20]	Egypt	2022	39/81	33 32 33	TAC vs. Verapamil vs. 5-Fu	24w	POSAS
13	Gamil HD [21]	Egypt	2019	34/16	19-43	TAC vs. BTA vs. BTA+TAC	6m	POSAS
14	Shaarawy E [22]	Egypt	2015	0/24	26.17 32.42	BTA vs. TAC	7m	POSAS
15	Margaret SFX [23]	India	2008	54	10-50	TAC vs. Verapamil	12m	VSS
16	Ahuja RB [24]	India	2014	40	15-60	TAC vs. Verapamil	24w	VSS
17	Srivastava S [25]	India	2018	20/20	30.45 29.45	TAC vs. Verapamil	24w	VSS
18	Chen XE [26]	China	2017	46	27.2 26.5	TAC vs. TAC+5-Fu	3m	POSAS
19	Liu Bin [27]	China	2017	51/39	18-53	BTA vs. TAC	6m	Effective rate
20	Wu Jiawen [28]	China	2019	58/32	30.18 29.76 29.57	TAC vs. BTA vs. BTA+TAC	12m	Effective rate
21	Gong Hui [29]	China	2021	31/8	40.70 40.94 34.52	TAC vs. BTA vs. BTA+TAC	6m	Effective rate, VAS, VSS
22	Zhu Ling [30]	China	2022	58/59	33.58 34.29	TAC vs. BTA+TAC	1m	Effective rate, VAS
23	Sudulagunta S [31]	India	2015	30/20	28.68 29.86	TAC+5-Fu vs. Bleomycin	12m	Scar height

*POSAS* Patient and Observer Scar Assessment Scale; *VSS* Vancouver Scar Score; *VAS* Visual Analog Scale

### Effective Rate

**Fig 2.**
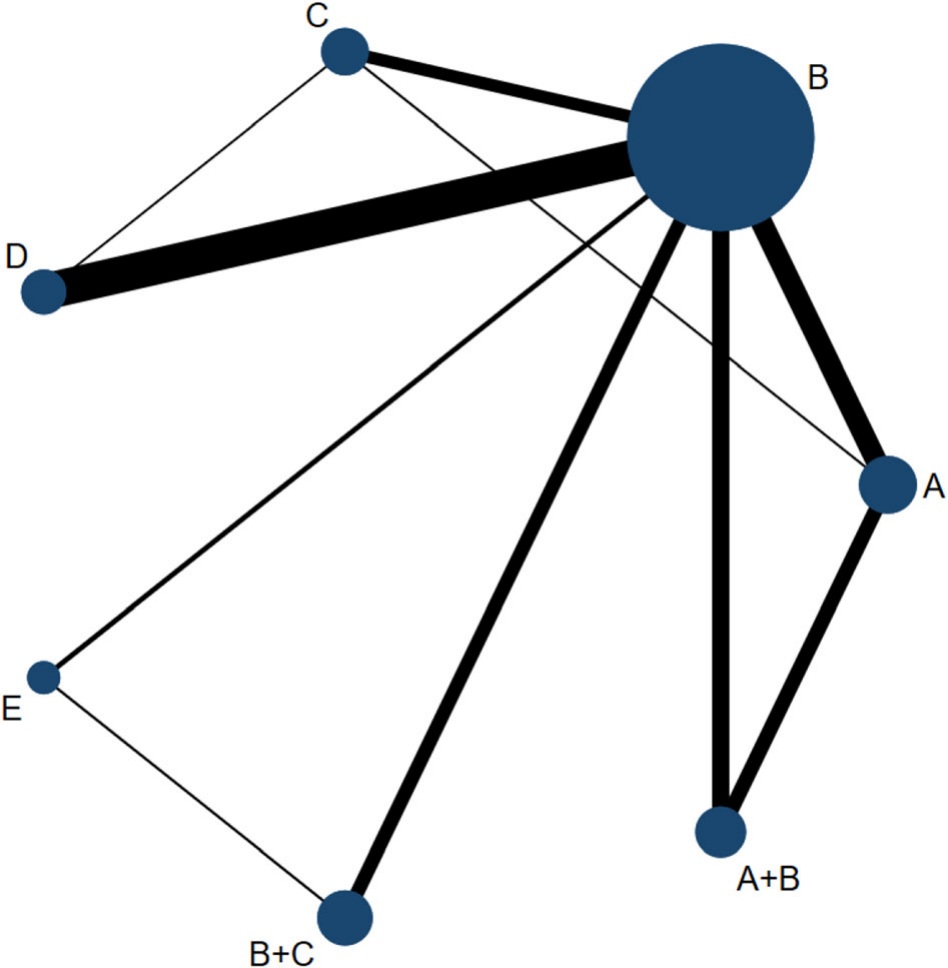
Network graph of effective rate. **A** BTA, **B** TAC, **C** 5-FU, **D** Verapamil, **E** Bleomycin

Five monotherapies and two combined measures were established for ten direct comparisons and eleven indirect comparisons. The monotherapies included A, BTA; B, TAC; C, 5-Fu; D, VER; or E, BLM. The network diagram is shown in Fig. 2, with each dot representing one treatment modality, the size of the dots indicates the sample size, and the wiring of the dots represents the two therapies with a direct contrast. The combination treatments included A+B, BTA combined with TAC, and B+C, TAC combined with 5-Fu.

### Publication Bias

**Fig 3.**
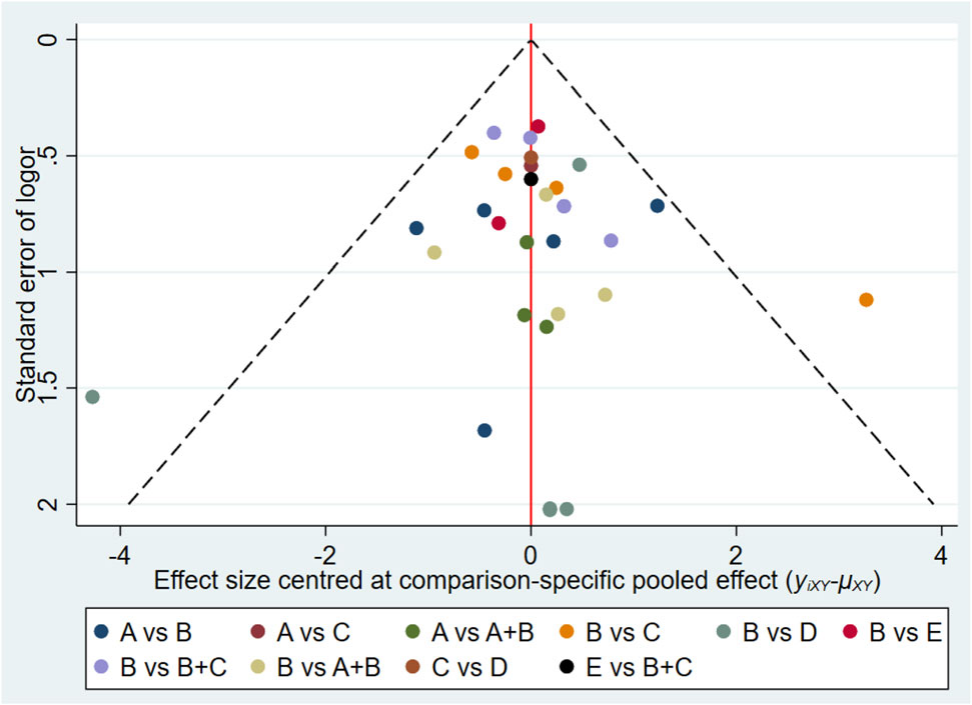
Funnel diagram. **A** BTA, **B** TAC, **C** 5-FU, **D** Verapamil, **E** Bleomycin

Publication bias in the literature was detected by the funnel plot, which was essentially symmetric, indicating no significant publication bias (Fig. 3).

### Network Meta-Analysis

Table 2 shows the key findings of this network meta-analysis. Compared to the effectiveness of the five monotherapies, the efficacy of BTA combined with TAC was significantly different from BTA alone, TAC alone, 5-Fu alone, VER alone, and BLM alone (BTA+TAC vs. BTA: RR=2.84, 95% CI, 1.13–7.12; BTA+TAC vs. TAC: RR=6.42, 95% CI, 2.88–14.31; BTA+TAC vs. 5-Fu: RR=10.25, 95% CI, 3.99–26.34; BTA+TAC vs. VER: RR=5.75, 95% CI, 1.81–18.29; BTA+TAC vs. BLM: RR=3.55, 95% CI, 1.31–9.57), but the efficacy of BTA+TAC was not significantly different from TAC+5-Fu (RR=0.55, 95% CI, 0.22–1.39).

**Table 2:** The network meta-analysis results

BTA	0.44 (0.24,0.82)	0.28 (0.14,0.56)	0.49 (0.18,1.36)	0.80 (0.34,1.88)	1.57 (0.73,3.38)	2.84 (1.13,7.12)
2.26 (1.22,4.21)	**TAC**	0.63 (0.36,1.08)	1.12 (0.48,2.61)	1.81 (1.01,3.26)	3.56 (2.28,5.57)	6.42 (2.88,14.31)
3.61 (1.79,7.29)	1.60 (0.93,2.74)	**5-Fu**	1.78 (0.75,4.23)	2.89 (1.30,6.42)	5.68 (2.82,11.47)	10.25(3.99,26.34)
2.03 (0.74,5.56)	0.89 (0.38,2.09)	0.56 (0.24,1.33)	**Verapamil**	1.62 (0.58,4.54)	3.19 (1.22,8.31)	5.75 (1.81,18.29)
1.25 (0.53,2.94)	0.55 (0.31,0.99)	0.35 (0.16,0.77)	0.62 (0.22,1.73)	**Bleomycin**	1.97 (1.01,3.84)	3.55 (1.31,9.57)
0.64 (0.30,1.37)	0.28 (0.18,0.44)	0.18 (0.09,0.36)	0.31 (0.12,0.82)	0.51 (0.26,0.99)	**TAC+5-Fu**	1.80 (0.72,4.52)
0.35 (0.14,0.88)	0.16 (0.07,0.35)	0.10 (0.04,0.25)	0.17 (0.05,0.55)	0.28 (0.10,0.76)	0.55 (0.22,1.39)	**TAC+BTA**

The efficacy of TAC+5-FU was better than that of TAC alone, 5-Fu alone, VER alone, and BLM alone (TAC+5-FU vs. TAC: RR=0.28, 95% CI, 0.18–0.44; TAC+5-FU vs. 5-Fu: RR=0.18, 95% CI, 0.09–0.36; TAC+5-FU vs. VER: RR=0.31, 95% CI, 0.12–0.82; TAC+5-FU vs. BLM: RR=0.51, 95% CI, 0.26-0.99), whereas there was no substantial distinction between the efficacy of TAC+5-FU and that of BTA alone (RR = 1.57, 95% CI: 0.73–3.38).

The therapeutic effectiveness of BTA by itself is superior to that of TAC alone and 5-Fu alone (BTA vs. TAC: RR=2.26, 95% CI 1.22–4.21; BTA vs. 5-Fu: RR=3.61, 95% CI, 1.79–7.29). However, the efficacy of BTA alone was not significantly different from that of VER alone and BLM alone (BTA vs. VER: RR=2.03, 95% CI, 0.74–5.56; BTA vs. BLM: RR=1.25, 95% CI, 0.53–2.94).

No statistically significant difference in effectiveness was found between TAC alone, 5-Fu alone, and VER alone (TAC vs. 5-Fu: RR=1.60, 95% CI, 0.93–2.74; TAC vs. VER: RR=0.89, 95% CI, 0.38–2.09; 5-Fu vs. VER: RR=0.56, 95% CI 0.23–1.33).

The therapeutic efficacy of BLM alone is superior to those of TAC alone and 5-Fu alone (BLM against TAC: RR=0.55, 95% CI, 0.31–0.99; BLM vs. 5-Fu: RR=0.35, 95% CI, 0.16–0.77), whereas BLM and VER alone were equally effective (BLM vs. VER: RR=0.62, 95% CI, 0.22–1.73).

### SUCRA Ranking Results

**Fig 4.**
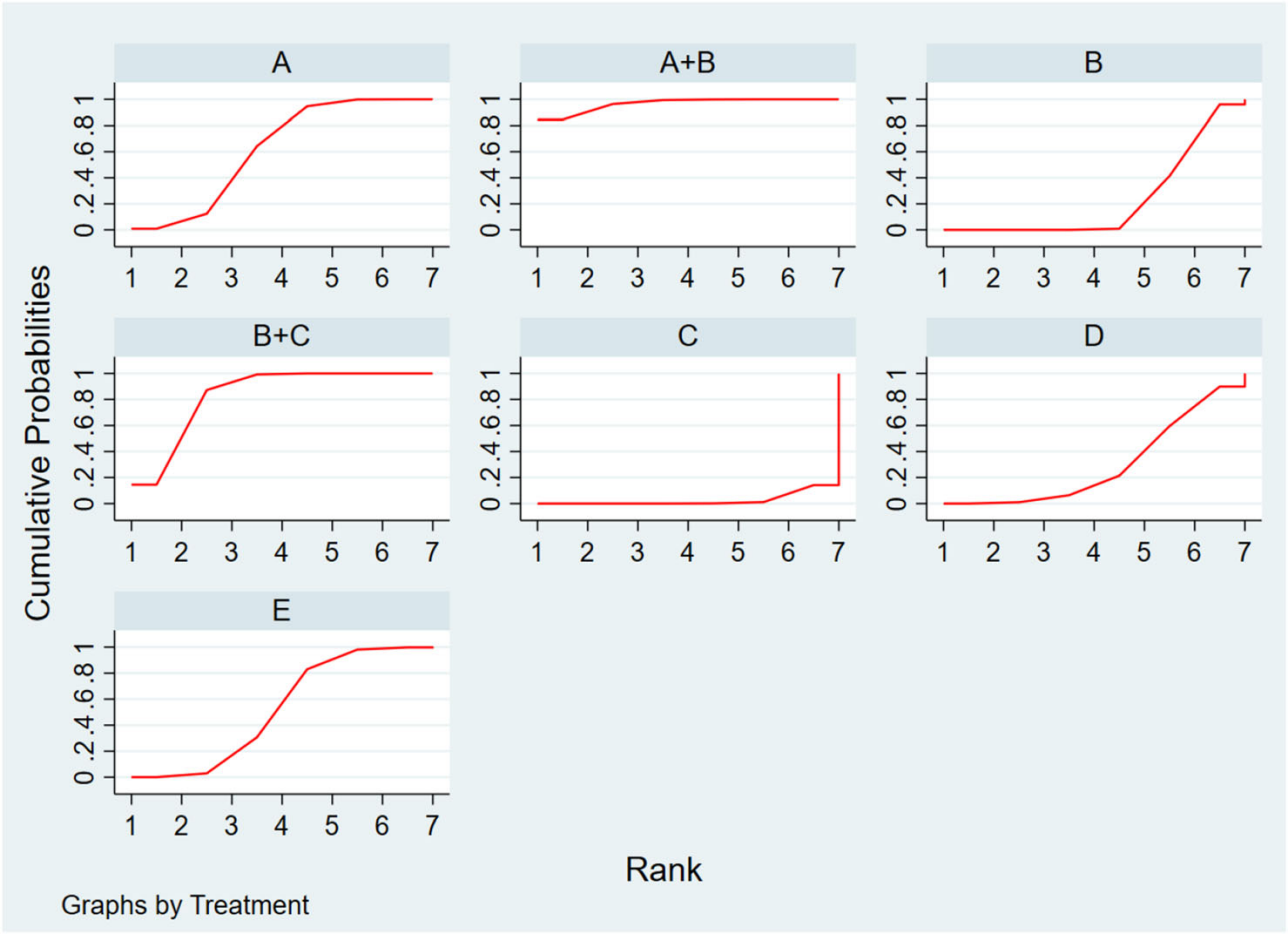
SUCRA efficacy rate ranking curve. **A** BTA, **B** TAC, **C** 5-FU, **D** Verapamil, **E** Bleomycin, SUCRA, surface under the cumulative ranking

The area under the curve was employed to estimate the probability that a given treatment would produce a cure, with a bigger area indicating greater efficacy. Seven therapies for keloids and hypertrophic scars were evaluated and ranked in order of effectiveness; TAC + BTA was the most effective (96.7%), followed by TAC + 5-Fu (83.5%), BTA (62.0%), BLM (52.4%), VER (29.7%), TAC (23.1%), and 5-Fu (2.6%) (Fig.4).

### Detection of Inconsistency

Twenty-three articles of seven treatment measures formed four triangular closed loops. The findings of this investigation indicated that *p* > 0.05 and the bottom 95% [CI] limits were 0, demonstrating that each loop's direct and indirect comparisons were consistent (Table 3).

**Table 3:** Inconsistency detection of closed loop for effective rate

Loop	*p* value	95% CI
TAC/5-Fu/verapamil	0.186	(0.00,6.67)
BTA/TAC/(BTA+TAC)	0.210	(0.00,3.39)
BTA/TAC/5-Fu	0.652	(0.00,3.24)
TAC/BLE/(TAC+5-Fu)	0.956	(0.00,1.47)

## Discussion

The medical community has long been interested in finding effective treatments for keloid and hypertrophic scars, and many drugs have achieved good results [7]. Commonly used drugs include BTA, TAC, 5-Fu, VER, and BLM [32], but most clinical studies have only conducted randomized controlled trials on two or three of them [19], so the efficacy of these drugs in treating pathological scars has been controversial [33]. Therefore, it is hoped that this network meta-analysis can provide some basis for plastic surgeons to choose the injected drugs for pathological scars.

According to the findings of this network meta-analysis, combination medications were more effective than monotherapy, with BTA+TAC and TAC+5-Fu being most effective for treating pathological scars. In most trials, BTA+TAC combination therapy demonstrated significant improvement in scar height and patient POSAS, VSS, and VAS.

BTA can not only act on the presynaptic membrane but also inhibits the release of acetylcholine [34] and the contraction of myofibroblasts, reducing the tension at the wound edge [35]. Related studies have provided evidence that BTA can regulate fibroblasts by multiple mechanisms [36], reducing scar formation, fibroblast proliferation, TGF expression, and collagen production, thereby inhibiting scar formation [37, 38].

TAC is not only a hormonal drug but also a well-known medication for dealing with scar disorders such as keloids and hypertrophic scars [39]. It prevents pathological scarring by reducing fibroblast growth, promoting collagen breakdown, and triggering apoptosis [40, 41]. Numerous studies indicate that TAC injection alone is less efficient with a high recurrence rate and more adverse reactions [42]; thus, it is not recommended for treating pathological scars.

5-Fu, an antitumor drug, can interfere with rRNA transcription, ultimately inhibiting fibroblasts and myofibroblast growth by inhibiting the deoxyribonucleic acid synthesis and reducing the expression of type I collagen [43, 44]. Interestingly, most studies dispute the efficacy of TAC alone and 5-Fu alone. However, when comparing TAC to 5-Fu for treating hypertrophic and keloid scars, this study demonstrated no significant difference in efficacy.

Verapamil is a calcium channel blocker, and later studies found that it could inhibit the proliferation of fibroblasts [45] and reduce the generation of the extracellular matrix and collagen [32]. It is worth noting that some scholars believe that the efficacy of TAC is better [46], and others believe that there is no difference in the efficacy of the two drugs [42, 47]. However, this network meta-analysis indicated that both medications treat hypertrophic scars and keloid well.

Since Bleomycin is a potent cytotoxic agent, it is widely used to treat several cancers [48]. Previous research has discussed the BLM mechanism of action in treating hypertrophic and keloid scars. BLM can hasten the process of apoptosis in fibroblasts and decrease the production of collagen, DNA, and RNA [44]. Up to now, the effectiveness of BLM in treating hypertrophic and keloid scars was only the subject of a limited number of systematic reviews and meta-analyses. Thus, further research regarding the effectiveness of BLM as a treatment for hypertrophic scars and keloids is crucial.

Even though there was no significant difference in the effectiveness of TAC+5-Fu and BTA+TAC, BTA+TAC was the most effective injection for treating pathological scars in the current study. However, there is limited clinical research evaluating the effectiveness of BTA+TAC in treating keloids and hypertrophic disorders. Consequently, the efficacy of BTA+TAC has significant implications for the selection of injectable medicines used in clinical treatment.

Some studies have proposed that combining TAC with 5-Fu to treat scars has better efficacy and fewer side effects than TAC alone [49]. Although the effectiveness of TAC+5-Fu was not different compared to BTA alone, it was significantly different compared to TAC alone, 5-Fu alone, VER alone, and BLM alone.

## Limitations

It should be acknowledged that this network meta-analysis has certain limitations. First, there are great differences in the usage and dosage of drugs between different clinical studies, which may lead to different research conclusions. Second, the follow-up duration of these RCTs was inconsistent, ranging from 12 weeks to 22 months; this may also lead to differences between trials. Third, the population included in the study had some regional and ethnic limitations. Fourth, we still cannot rule out the possibility that the conclusions were influenced by publication bias. Last, our study was not investigated the other drugs.

## Conclusions

According to the network meta-analysis results and the SUCRA sequence diagram, combination therapy, particularly BTA+TAC, is recommended for managing pathological scars. However, the limitations of this study's findings necessitate additional clinical research to confirm the conclusions.
